# Editorial: Anti-cancer and anti-neurodegenerative activities of nutraceuticals

**DOI:** 10.3389/fnut.2023.1242145

**Published:** 2023-07-12

**Authors:** Rong Li

**Affiliations:** Key Laboratory of Environmental Pollution and Integrative Omics, Education Department of Guangxi Zhuang Autonomous Region, Guilin Medical University, Guilin, China

**Keywords:** anti-cancer, anti-neurodegenerative, nutraceuticals, transcriptome, vitamins

Nutraceuticals are products based on the nutrient content of foods derived from dietary or medicinal plants (1). Because of their ability to modulate different molecular and cellular pathways, nutraceuticals are considered beneficial for preventing and treating cancers and neurodegenerative diseases (2, 3). Furthermore, nutraceuticals' presumed safety and potential nutritional and chemopreventive activities have generated significant global interest in identifying novel nutraceuticals. According to some epidemiological studies, an appropriate nutritional supplement can prevent 35% of cancer-related deaths, and 90% of cancers can be prevented by dietary supplementation (4). Accumulating evidence strongly suggests that nutraceuticals can prevent the onset of neuronal damage and subsequently reduce the progression of neuronal destruction (5). In recent years, many scientists have focused on using nutraceuticals to prevent cancer and neurological diseases through different mechanisms, including antioxidant and anti-inflammatory effects, mitochondrial homeostasis, autophagy regulation, and the promotion of neurogenesis. However, detailed molecular mechanisms underlying these protective effects remain largely unknown. The current Research Topic is issued in the Clinical Nutrition section of the Frontiers in Nutrition journal, including four original research articles. We would like to summarize the issue in the following two major directions.

## Anti-neurodegenerative activities

Luo et al. used a middle cerebral artery occlusion rat model and comparative transcriptomics to determine the molecular targets and mechanisms underlying the beneficial effects of laminarin in ischemic stroke (Luo et al.). Their results demonstrated the involvement of laminarin targets in blood circulation, oxygen supply, and anti-inflammatory responses in the normal brain. More importantly, laminarin treatment attenuated the brain damage and neuro-deficits caused by an ischemic stroke. Using bioinformatics analysis, they found that the beneficial effects of laminarin occur through the regulation of blood vessel development and brain cell death, suggesting that laminarin could be a novel nutraceutical for treating ischemic stroke.

## Anti-cancer activities

Vaughan-Shaw et al. performed a feasibility study to evaluate whether high-dose cholecalciferol (vitamin D3) had a beneficial effect on the survival of patients with CRC (Vaughan-Shaw et al.). We recruited 122 colorectal cancer (CRC) patients, 41 of whom were administered high-dose vitamin D3 daily. Serum 25-hydroxyvitamin (25OHD) levels were measured using liquid chromatography-tandem mass spectrometry and compared with untreated CRC controls. They found that high-dose vitamin D supplementation was associated with higher perioperative 25OHD levels, lower rates of vitamin D insufficiency, and reduced early postoperative C-reactive protein levels. These findings suggested that vitamin D has beneficial effects on CRC survival outcomes.

Another prospective cohort study conducted by Temraz et al. investigated the prognostic value of vitamin C in patients with mCRC, recruiting adults with metastatic CRC (mCRC) (*n* = 46) and cancer-free controls (*n* = 45) (Temraz et al.). They found no significant difference in vitamin C intake between the two groups; however, patients have lower plasma vitamin C levels than healthy controls.

Sannappa Gowda et al. used an *in vivo* mouse mammary carcinoma model and an *in vitro* human breast cancer cell line model to study the hepatoprotective role of quercetin in breast cancer via the vitamin D receptor (VDR) (Sannappa Gowda et al.). Their results showed that quercetin treatment significantly decreased tumor volume, tumor angiogenesis, hepatic inflammation, and fibrosis. Furthermore, the results of the docking analysis confirmed the VDR-quercetin interaction, suggesting the involvement of VDR in anti-breast cancer activity.

## Conclusion

In conclusion, this issue suggests using nutraceuticals such as vitamin D3 and laminarin to treat cancers and neurodegenerative diseases. Several studies have delineated the molecular mechanisms underlying the beneficial effects of these nutraceuticals. However, further preclinical studies are required to confirm these findings before the clinical use of these nutraceuticals.

## Author contributions

The author confirms being the sole contributor of this work and has approved it for publication.
